# Video-Assisted Anal Fistula Treatment: Pros and Cons of This Minimally Invasive Method for Treatment of Perianal Fistulas

**DOI:** 10.1155/2017/9518310

**Published:** 2017-06-07

**Authors:** Michal Romaniszyn, Piotr Walega

**Affiliations:** 3rd Department of General Surgery, Jagiellonian University Medical College, Kraków, Poland

## Abstract

**Purpose:**

The purpose of this paper is to present results of a single-center, nonrandomized, prospective study of the video-assisted anal fistula treatment (VAAFT).

**Methods:**

68 consecutive patients with perianal fistulas were operated on using the VAAFT technique. 30 of the patients had simple fistulas, and 38 had complex fistulas. The mean follow-up time was 31 months.

**Results:**

The overall healing rate was 54.41% (37 of the 68 patients healed with no recurrence during the follow-up period). The results varied depending on the type of fistula. The success rate for the group with simple fistulas was 73.3%, whereas it was only 39.47% for the group with complex fistulas. Female patients achieved higher healing rates for both simple (81.82% versus 68.42%) and complex fistulas (77.78% versus 27.59%). There were no major complications.

**Conclusions:**

The results of VAAFT vary greatly depending on the type of fistula. The procedure has some drawbacks due to the rigid construction of the fistuloscope and the diameter of the shaft. The electrocautery of the fistula tract from the inside can be insufficient to close wide tracts. However, low risk of complications permits repetition of the treatment until success is achieved. Careful selection of patients is advised.

## 1. Introduction

Perianal fistula is one of the oldest gastrointestinal conditions known in medicine. Despite ages of research and development, the precise causes of this disease in its most common; cryptoglandular variants are still unknown. It is also a common problem for patients with the anorectal form of Crohn's disease. For most cases, the only treatment modality is surgery. Although the fistulotomy/fistulectomy procedure is still considered the “gold standard” of treatment, nevertheless, the risk of serious complications remains an issue. Some measure of fecal incontinence continues to affect 10%–45% of patients operated upon [1, 2], with success rates varying from 70% to 90% [3].

Published studies have shown that complex, branched, or recurrent fistulas are at a higher risk of treatment failure and complications [4]; therefore, a careful diagnostic evaluation is needed to avoid pitfalls. The two most common diagnostic tests performed are endoanal ultrasound (EUS) with hydrogen peroxide administered to the lumen of the fistula and magnetic resonance imaging (MRI) of the pelvis. Unfortunately, even these methods lack the sensitivity required to fully assess the exact course and form the fistula tract [5, 6]. Moreover, intraoperative exploration of the fistula tract with a simple, rigid proctological probe can lead to creation of a false tract in perianal tissues and transform a simple fistula into a complex one.

In many fields of surgery, videoscopic, minimally invasive procedures are becoming increasingly more popular. Therefore, in 2006, a special fistuloscope was created by Dr. Piercarlo Meinero [7]. This minimally invasive device allows endoscopic treatment of perianal fistulas under direct visual guidance. The procedure consists of two phases: a diagnostic phase and an operative phase, performed subsequently. During the diagnostic phase, the fistuloscope is inserted through the external opening, with fluid perfusion which permits direct vision of the fistula tract. The fistula tract and its branches are then explored, and the internal opening is identified by direct vision or fluid flow. During the operative phase, the fistula tract and all its branches are destroyed under direct vision, using a cautery electrode. The necrotic remnants are removed with an endo brush or Volkmann spoon, and the wound flushed with perfusion fluid. The internal opening is then closed with a stapler (original method), sutures, or an advancement flap [8]. The initial results published in 2011 were very promising [7], but in the publications which followed the results, it varied significantly [9–11].

## 2. Purpose

The purpose of this paper is to present results of a single-center, nonrandomized, prospective study of the video-assisted anal fistula treatment.

## 3. Methods

The study was designed as a nonrandomized, prospective observational study. All consecutive patients with a perianal fistula who qualified for elective surgery during the years 2011–2016 were enrolled in the study. The only exclusion criterion was patients with low intersphincteric fistulas (treated by the simple “lay-open” procedure) or patients who refused to undergo the minimally invasive treatment with the use of a fistuloscope. In total, 68 patients (48 males and 20 females) were enrolled and underwent the VAAFT procedure. IBD was not an exclusion criteria, but by chance, no patients with Crohn's disease enrolled. The mean age was 43.8 years (24–81 years). There was no preselection of patients, and all were qualified for fistuloscopy by default (unless they met any of the aforementioned exclusion criterion). The fistulas were not routinely prepared by seton insertion preoperatively; however, abscesses or larger fluid collections were drained before qualification for the procedure. The patients were not examined with MRI or EUS upon qualification; however, some patients had an MRI or EUS examination earlier during their treatment. The results of these examinations were not taken into consideration during the enrollment process. None of the patients complained of any continence disorders before the procedure. The chief complaints were pain in the anal region, excretion of pus, and occasional bleeding from the external opening.

**Figure 1:**
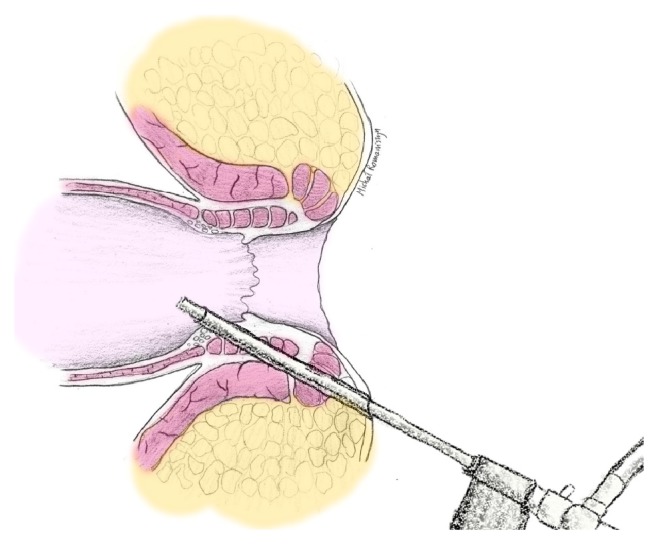
Schematic of a fistuloscope inserted into the fistula tract, with identification of the internal opening.

The VAAFT procedure was carried out using spinal analgesia, with a single dose of antibiotic prophylaxis (cephazolin, metronidazole). The patients were positioned in the lithotomy position. The fistuloscope (Karl STORZ, GmbH) was then introduced into the external opening (Figure 1), and the VAAFT procedure was performed according to the description of Meinero and Mori [7], except for the closure of the internal opening, which was performed with either a “figure of eight” suture (65 patients) or an advancement flap (3 patients), rather than by means of a stapler. The tracts were destroyed by means of electrocautery, the necrotic tissues removed and the external openings were cored out and left open for drainage. The patients were discharged the day following the procedure. Patients were recommended a high-fiber diet and to flush the wound with an antiseptic once daily, and following each bowel movement. No antibiotic therapy was administered after the procedure. The patients were prescribed 500 mg of paracetamol QID for pain control, if necessary. The patients were followed up as needed, until reaching an endpoint (either complete healing or the wound having a continuous discharge with no prognosis of healing-persistent fistula).

Persistence of fistula was defined as an unhealed wound with constant discharge, whereas recurrence was defined as reopening of a previously healed fistula tract or formation of an abscess after complete closure of the wound. In cases of a recurrent or persistent fistula, the patients were treated based on clinical evaluation.

The data was analyzed using StatSoft® STATISTICA® software, with nonparametric tests (Mann–Whitney *U* test, Spearman's ANOVA, correlation matrices) and cross tabulation tests (Pearson's chi^2^).

## 4. Results

**Figure 2:**
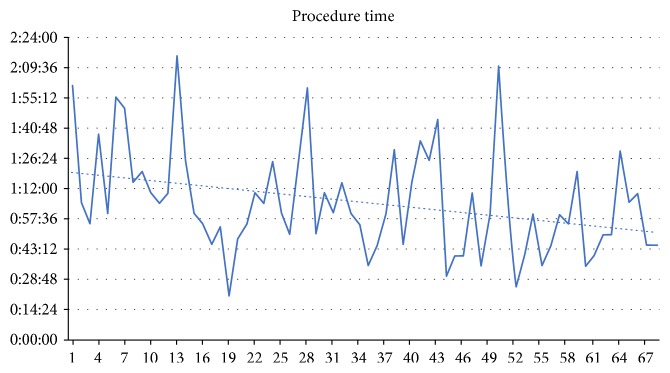
Operating time case by case—correlation with learning curve.

The mean follow-up was 31 months (3–72 months, median: 26 months). There were 30 patients with simple transsphincteric fistulas, and 38 patients who had complex fistulas (branched, with multiple openings, extrasphincteric or suprasphincteric, with collections of fluid in the soft tissues, etc.). On average, operating time was 65 minutes (20–135 minutes), and there was a correlation with a drop in operating time and the learning curve (Figure 2). Of the 68 patients, 51 (75%) achieved primary healing. On average, it took 52 days (15–98 days) for the wound to heal. The remaining 17 patients (25%) never healed after the VAAFT procedure and were eventually qualified for secondary procedures (repeated VAAFT, fistulectomy, or seton). In the group which initially healed, there were 14 cases of fistula recurrence (20.59% of the entire group of 68 patients). The recurrences took place between 1 and 6 months after the initial healing, with two extraordinary cases of recurrence 23 and 38 months after initial healing. The overall healing rate of the VAAFT procedure was 54.41%—37 of the 68 patients healed and had no recurrence in the follow-up period.

**Figure 3:**
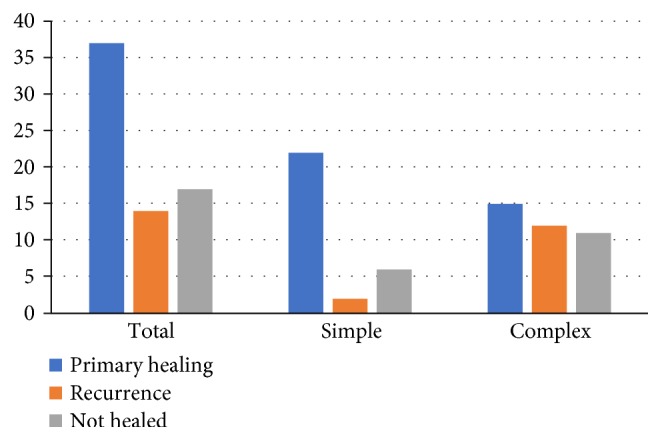
Results of treatment depending on the type of fistula.

The results of the procedure varied depending on the type of fistula. Therefore, during data analysis, two subgroups were distinguished—simple transsphincteric and complex fistulas. In the simple transsphincteric group, 24 of the 30 patients healed (80%). Two of the patients (6.67%) had recurrence of the fistula after their initial healing, while the remaining 22 patients healed without recurrence. The fistulas of 6 of the 30 patients in this group (20%) never healed after the VAAFT procedure. The eventual, overall success rate in this group with simple transsphincteric fistulas was 73.3% (Figure 3).

However, the other group, with complex perianal fistulas, had lower success rates. Of the 38 patients in this group, only 15 healed without recurrence (39.47%), 12 healed but recurred (31.58%), and 11 had persistent fistula after the VAAFT (28.95%) (Figure 3). The difference in the success rates between the group with simple and the group with complex fistulas (73.3% versus 39.47%) was statistically significant (Pearson's chi^2^*p* = 0.011). Summary of these results is shown in Table 1.

**Figure 4:**
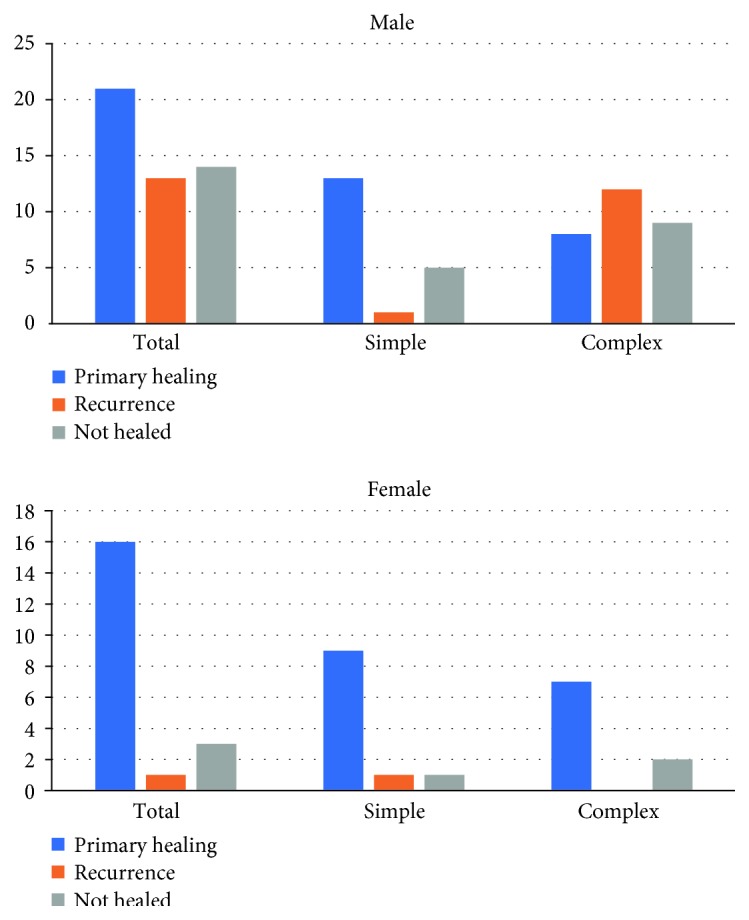
Results depending on gender and type of fistula.

One interesting observation was that female patients achieved higher healing rates both in simple transsphincteric (81.82% versus 68.42%, although Pearson's chi^2^*p* = n.s.) and complex fistulas (77.78% versus 27.59%, Pearson's chi^2^*p* = 0.016) (Figure 4). The patients' age had no influence on the results (*p* = n.s.).

Two adverse events took place in the study group. One patient suffered from severe headaches associated with the spinal analgesia, although the symptoms resolved after treatment with oral painkillers. In another patient, the cautery probe was damaged during the surgery (by an electric arch), leaving a severed metal electrode tip in the wound. The tip was successfully recovered with forceps during the same procedure. There were no other complications associated with the VAAFT procedure. None of the patients reported any worsening of continence after surgery.

## 5. Discussion

**Figure 5:**
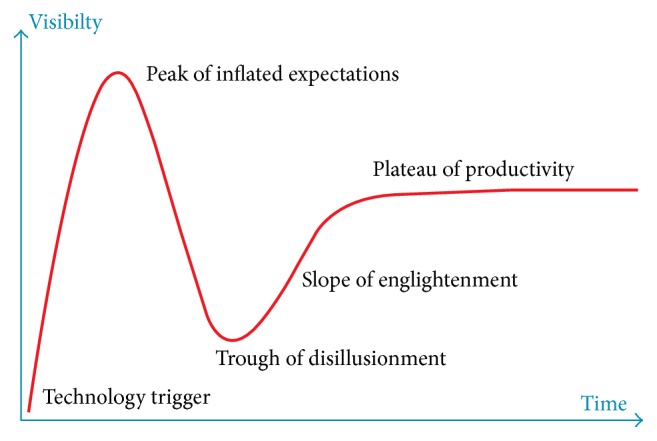
Gartner's hype cycle (adapted from http://www.gartner.com/technology/research/methodologies/hype-cycle.jsp).

Each time a new technique emerges, there is an initial enchantment among researchers and practitioners alike. However, interest often changes with time, as further research and a longer follow-up period reveal the pros and cons of the new technique. After a period of critical appraisal, the new technique either finds its place among other modalities or is considered inadequate and perishes. This process is common not only for medical but for most technological research and usually follows the Gartner's hype cycle (Figure 5). Apart from the fistulectomy, which is considered the “gold standard” of treatments, and has up to a 90% healing rate, there are several techniques which have withstood the “hype cycle” challenge. One is the LIFT technique (ligation of intersphincteric fistula tract), which has success rates ranging from 39.8% [12] to about 82% [13], with vast differences in results of the LIFT technique reported by different authors [14]. Other techniques, such as fistula plugs, have been verified by several studies and proved to not be as efficient as was initially estimated [15].

### 5.1. State of the Art

Initial results of this new minimally invasive treatment of perianal fistulas were optimistic, ranging from 73% up to 92% of success rates for short-term follow-up [7, 8, 16]. As more and more research centers published their results, the effectiveness of the procedure started to be more variable, dropping closer to 67%–71% [10, 11, 17]. Moreover, a closer look at the published results revealed either short follow-up (Meinero and Mori) [7], some form of bias, such as patient preselection for the procedure, based on clinical or MRI evaluation prior to the operation (Chowbey et al. and Kochhar et al.) [9, 16], or creation of diverting stoma in some patients (Schwandner) [18]. Obviously, there have been no randomized trials on the VAAFT procedure as of yet.

Furthermore, soon after Meinero's publication, several comments appeared [19, 20], pointing out potential flaws of endoscopic treatment using the fistuloscope. Parks et al. noted that perianal fistulas come in various shapes and sizes, and due to anatomical construction and the relations of pelvic structures, the tract is often curved [21]; therefore, it is often difficult to explore the entire tract of the fistula using a rigid instrument. This is in accordance with our experience, as we found the suprasphincteric fistulas particularly difficult to explore if there is a tight corner right before the internal opening. Moreover, the diameter of the fistuloscope's shaft makes narrow tracts, with fibrous, stiff walls inaccessible for the device. Careful exploration with a cautery electrode inserted through the working canal of the fistuloscope may enable further exploration of the narrowed tract; however, the advantage of visual guidance is lost.

### 5.2. Study Result Analysis

Our study showed that there is a significant difference between results of the minimally invasive treatment of simple transsphincteric and complex fistulas. Complex fistulas are defined in most publications as fistulas consisting of multiple tracts, involving more than 30% of the external sphincter, recurrent fistulas, or those associated with preexisting fecal incontinence, inflammatory bowel disease, or radiation [22, 23]. These fistulas are certainly more difficult to heal [24] and involve higher risk of fecal incontinence in up to a shocking 66% of patients [25]. The vast difference between the efficacy of the VAAFT in simple and complex fistulas (73.3% versus 39.47%) may be responsible for the variability of success rates reported by different authors. The overall healing rate probably depends on the structure of the study group—the more patients with simple transsphincteric fistulas, the better the overall results. A notable exception from this observation is a paper by Schwandner, which concentrated on patients with complex perianal fistulas of patients with Crohn's disease and achieved acceptable results (82%). However, some patients had diverting stoma, the follow-up was rather short, and the group was small [18]. Failure of minimally invasive treatment of the complex fistulas may not only be due to difficulties in adequate exploration of multiple or curved tracts and proper identification of the internal opening but also because of the diameter of the fistula tract and larger collections of fluid. Electrocautery of the fistula tract from the inside may not be sufficient to close tracts which are large in diameter. Some experts also note that excessive ablation of fistula tracts may cause collateral thermal damage to the tissues lying outside the area of fistula granulation [20].

Apart from the “simple-to-complex fistulas” ratio in the study group, the “male-to-female” ratio also seems to play a role, as women in our study group achieved significantly better healing rates than men. Although most published papers on fistula treatment state that perianal fistula is more common in men than that in women [24], the healing rate differences between both genders have not been analyzed in the literature; therefore, it is difficult to explain this observation, but it definitely requires further confirmation on larger groups of patients.

### 5.3. Advantages of VAAFT

It is worth noting that it seems that endoscopic treatment of perianal fistulas is not associated with any major complications. Apart from our report of two minor adverse events, there have been a few minor complications reported by several authors—urinary retention and perineal or scrotal oedema [7, 9]. Therefore, the VAAFT procedure may be viable in patients with complex perianal fistulas due to its low risk of complications, as generally the procedure can be safely repeated until success is achieved. The video fistuloscope gives the operating surgeon greater control over the procedure, as visualization of the tract and its branches helps to identify the exact course of the fistula and its internal opening. This helps to avoid creation of a false tract or false internal opening while blindly and forcefully exploring the tract with a fistula probe. Moreover, the visual guidance helps to identify side branches of the fistula, which would otherwise be unobserved and omitted.

### 5.4. Disadvantages of VAAFT

Video-assisted anal fistula treatment has a lower success rates than the “gold standard” fistulotomy which is an obvious disadvantage of this technique. Apart from the overall results, there are some technical aspects, which may be responsible for the failure of the procedure, in certain patients. Due to the instrument's construction, adequate exploration of multiple or curved tracts and proper identification of an internal opening may be difficult, as it is sometimes not possible to lead the rigid shaft of the fistuloscope through any sharp curves of the tract. Also, the fistula tract must be wide enough to let the fistuloscope through, but narrow enough for the electrocautery to be effective, as wide tracts or collections render the cauterization ineffective. Furthermore, excessive cauterization may cause collateral thermal damage to tissues lying outside the area of fistula.

## 6. Conclusions

The results of this minimally invasive treatment vary greatly, depending on the type of fistula; therefore, results of other clinical studies on the VAAFT procedure must be analyzed in context of the study group's structure (ratio of patients with simple and complex fistulas).

In our study, female patients achieved better results than male—this observation needs to be confirmed in larger groups of patients.

There are some drawbacks of the procedure, due to the rigid construction of the fistuloscope and the diameter of the shaft. Moreover, the electrocautery of the fistula tract from the inside may not be sufficient to close tracts which are large in diameter.

On the other hand, low risk of complications allows repetition of the minimally invasive treatment, until success is achieved. Proper selection of patients is advised to balance success rates, cost-effectiveness, and the potential risk of complications for each patient.

## Figures and Tables

**Table 1:** Summary of results.

Fistula type	*n*	Primary healing	Recurrence	Failed to heal	Overall healing rate
Simple transsphincteric	30	24 (80.00%)	2 (6.67%)	6 (20.00%)	22 (73.30%)
Complex	38	27 (71.05%)	12 (31.58%)	11 (28.95%)	15 (39.47%)
Total	68	51 (75.00%)	14 (20.59%)	17 (25.00%)	37 (54.41%)
